# Supplementary material for: Complexome profiling of the Chlamydomonas psb28 mutant reveals TEF5 as an early PSII assembly factor
Source: Plant Cell. 2025 Mar 17;37(6):koaf055. doi: 10.1093/plcell/koaf055 (PMC12164590; doi:10.1093/plcell/koaf055)
Supplement: koaf055_Supplementary_Data [file koaf055_supplementary_data.zip › psb28_interactive.zip!/plots/intensity/qualityPlots/peptides/_Cre11.g468400.t1.1.html]

### Cre11.g468400.t1.1

Trivial name:   
SeqCoverage [median]: 6.90  
Mass: 53.29 kDa  
  
 

| Sequence | MissedCleavage | Mass [Da] | wtIntensity | muIntensity | Score |
| --- | --- | --- | --- | --- | --- |
| AYAAFGALAEAEAAK | 0 | 1452.72 | 0.00 | 2134.17 | 70.49 |
| NMQDESEPVSVVK | 0 | 1460.68 | 0.00 | 600.52 | 94.62 |
| VQVAMLER | 0 | 944.51 | 330.95 | 0.00 | 86.95 |
|  |  |  | 331.0 | 2734.7 |  |
